# Evolutionary history of sickle-cell mutation: implications for global genetic medicine

**DOI:** 10.1093/hmg/ddab004

**Published:** 2021-01-18

**Authors:** Kevin Esoh, Ambroise Wonkam

**Affiliations:** Division of Human Genetics, Department of Pathology, University of Cape Town, Cape Town, South Africa; Division of Human Genetics, Department of Pathology, University of Cape Town, Cape Town, South Africa; Department of Medicine, Faculty of Health Sciences, University of Cape Town, Cape Town, South Africa

## Abstract

Resistance afforded by the sickle-cell trait against severe malaria has led to high frequencies of the sickle-cell mutation [*HBB*; c.20T>A, p.Glu6Val; OMIM: 141900 (*HBB-β^S^*)] in most parts of Africa. High-coverage sequencing and genotype data have now confirmed the single African origin of the sickle-cell gene variant [*HBB*; c.20T>A, p.Glu6Val; OMIM: 141900 (*HBB-β^S^*)]. Nevertheless, the classical *HBB-*like genes cluster haplotypes remain a rich source of *HBB-β^S^* evolutionary information. The overlapping distribution of *HBB-β^S^* and other disease-associated variants means that their evolutionary genetics must be investigated concurrently. In this review: (1) we explore the evolutionary history of *HBB-β^S^* and its implications in understanding human migration within and out of Africa: e.g. *HBB* haplotypes and recent migration paths of the Bantu expansion, occurrence of ~7% of the Senegal haplotype in Angola reflecting changes in population/SCD dynamics, and existence of all five classical *HBB* haplotype in Cameroon and Egypt suggesting a much longer presence of *HBB-β^S^* in these regions; (2) we discuss the time estimates of the emergence of *HBB-β^S^* in Africa and finally, (3) we discuss implications for genetic medicine in understanding complex epistatic interactions between *HBB-β^S^* and other gene variants selected under environmental pressure in Africa e.g. variants in *HBB*, *HBA*, *G6PD*, *APOL1*, *APOE*, *OSBPL10* and *RXRA.*

## Introduction

Sickle-cell disease (SCD) is a group of blood disorders caused by mutations in *HBB* that promote haemoglobin (Hb) polymerization and sickling of red blood cells. The most common and most clinically severe form of SCD is sickle-cell anaemia (SCA, MIM: 603903), caused by homozygosity of the sickle-cell gene variant [*HBB*; c.20T>A, p.Glu6Val; OMIM: 141900 (*HBB-β^S^*)], or the co-inheritance of *HBB-β^S^* and β^0^-thalassaemia. Although the *HBB-β^S^* is estimated to have originated more than 7000 years ago (1), with a high recessive lethality, and excess mortality of 50–90%, it has persisted in appreciable frequencies because of the protection that heterozygotes have against severe *Plasmodium falciparum* malaria. Nevertheless, the classical *HBB-*like gene cluster haplotypes remain a rich source of information for variable SCD clinical expressions, and for interpreting regional and global human migrations. An estimated 300 000 babies are born each year with SCD worldwide, with nearly 75% of the births occurring in sub-Saharan Africa (SSA) (2). Therefore, as global population expansion and migration rates are predicted to double in the next three decades (3), it is expected that the *HBB-β^S^* frequency will increase and reach places previously naïve to it. Moreover, increased awareness, newborn screening programs and comprehensive care including emerging therapeutics and curative interventions will also contribute to increased SCD survival, and the *HBB-β^S^* variant globally, urging for the need to understand the historical distribution of the gene in an evolutionary context.

There is considerable overlap in the geographical distribution between *HBB-β^S^* and other gene variants that are known to have been adaptively selected in African genomes to protect against malaria, such as the thalassaemias (alpha− and beta−), and G6PD deficiency variants. Similar overlap exists between *HBB-β^S^* and gene variants adaptively selected against other infectious comorbidities such as the African trypanosomiasis, i.e. variants in *APLO1*. Therefore, concurrent co-inheritance of *HBB-β^S^* and other gene variants in African individuals could result in complex gene–gene (epistatic/non-allelic and allelic), and gene–environment interactions that may pose specific challenges or hold specific benefits that are yet to be fully investigated. In this review, we (1) explored the evolutionary genetic history of *HBB-β^S^* and its implications in understanding human migration within and out of Africa, (2) reviewed and discussed the time estimates of emergence of the *HBB-β^S^* variants in Africa and finally, (3) discussed perspectives in understanding complex epistatic interactions between the *HBB-β^S^* and other gene variants adaptively selected under environmental pressure in Africa (e.g. variants in *HBB*, *HBA* (OMIM: 141800), *G6PD* [OMIM: 305900] and *APOL1* [OMIM: 603743]), and their implications for genetic medicine for African populations, and beyond.

## Search Strategy and Selection Criteria

The global georeferenced database of HbS data (1950–2015) was retrieved and complemented by electronic database searches for full article published between—2020 using the search strategies below described in the supplementary material and summarized in Supplementary Material, Figure S1.

## Common Variants in Haemoglobin Genes

Over 3000 haemoglobin variants have been reported (https://www.ithanet.eu/db/ithagenes), several of which either affect qualitatively haemoglobin structure (haemoglobinopathies) or quantitatively haemoglobin expression level (thalassaemias). A plethora of *HBB* genes haemoglobinopathies exist, of which the *HBB-β^S^* (*HBB*; c.20T>A, p.Glu6Val; OMIM: 141900), HbC (*HBB*; c.19G>A, p.Glu6Lys; OMIM: 141900) and HbE (*HBE1*; c.79G>A, p.Glu26Lys; OMIM: 142100) are the most clinically significant (4,5). Alpha thalassaemias (OMIM: 604131) result mostly from the deletions at least one of the four alpha-globin genes (*HBA,* OMIM: 141800) and rarely from point mutations, whereas beta-thalassaemias (OMIM: 613985) are mostly caused by single nucleotide polymorphisms (SNPs) in the *HBB* gene (6). Thalassaemias major (α^0^/β^0^) involve the complete absence of the alpha-/beta-globin chains, and are usually clinically severe, with associated public health implications, whereas thalassaemia intermedia (α^+^/β^+^), minor or carrier, involving decreased production of the globin chains, are usually benign (7).

### Global distribution of the *HBB-β^S^* variant

The evolutionary interaction between *HBB-β^S^* and malaria, that has persisted for at least 5000 years, is perhaps the most characterized selection force in the human genome. The evolutionary link between *HBB* gene cluster variants and malaria—known as the ‘malaria hypothesis’—was postulated in 1949 by Haldane (8) and confirmed in 1954 by Allison based on *β^S^* data from Uganda and Kenya (9). Linkage analysis has shown that human genetic factors contribute up to 25% in severe malaria phenotypic variations, and that the sickle-cell trait (SCT), the heterozygous state of *HBB-β^S^* variant, exerts the strongest monogenic effect, albeit only 2% (10). Indeed, as Haldane noted, only a small gain in fitness would be required for a variant to attain its equilibrium frequency (8). The strongest SCT protective effect has now been recorded in West African (Gambia), whereas the weakest effect was recorded in Cameroon, although it is unclear whether fine-scale genetic sub-structure in Cameroon may have confounded these analyses (11,12).

The gain in fitness afforded by the SCT has resulted in the overlapping geographic distribution of the *HBB-β^S^* variant with the historical malaria endemicity regions (13). Generally, equatorial regions, particularly the stretch from western Ghana, through West-Central Africa and down to the north of the Zambezi, with historical malaria holo- and hyper-endemicity (year out malaria exposure and reinfections) harbour near-equilibrium frequencies of *HBB-β^S^* suggesting that the mutation has been around this region longer than anywhere else (13,14). The low prevalence of the *HBB-β^S^* in northern Africa, its virtual absence from indigenous populations in southern Africa, as well as in the horn of Africa (Ethiopia, Eritrea, Somalia and Djibouti), reflects a low *P. falciparum* malaria selective pressure in these areas. The distribution of the *HBB-β^S^* outside of Africa greatly reflects historical regional migrations from Africa to the Mediterranean, the Middle East, and the Indian sub-continent regions, and much recently trade routes such as the trans-Sahara slave trade (involving northern Africa and the Persian Gulf—the Arab World), the Islamic and European slave trades across the Nile and the Indian Ocean (involving the Arabian peninsula, India and South East Asia), and the trans-Atlantic slave trade (involving West-Central Africa, the Americas and Europe) (15). The East African slave trade dates, back to the 7th century and involved mostly women taken as domestic and sex slaves, hence the high prevalence of the *HBB-β^S^* gene in Middle Eastern and South Asian indigenous populations. Although the trans-Atlantic slave trade only effectively took off in the 16th century and involved mostly men taken as plantation workers, it is believed to have involved, by far, more slaves than the East African slave trade. Malaria selective environment in these regions further augmented the gene frequencies. Recent migrations have also contributed to the prevalence of the gene in regions with low or no malaria prevalence, for instance in southern Africa (16), North America (3) and Europe (17).

Interestingly, patches of regions within Central and West Africa, notably south of Liberia, surrounded by regions with high malaria endemicity, display much lower frequencies of *HBB-β^S^* (18). In some of these regions, the *HBB-β^S^* prevalence is offset by the opposing *HBB-*HbC variant such as in Burkina Faso, southern Mali and Northern Ghana where it is most prevalent, reaching frequencies > 15% (19). The homozygous HbCC affords a stronger protection against severe *P. falciparum* malaria than the HbAC and HbAS (20). Therefore, a positive directional selection is favouring the *HBB-*HbC variant to eventually replace the *HBB-β^S^* in malaria endemic regions. However, it has been observed that the rate of increase of the *HBB-*HbC variant is slower than that of the *HBB-β^S^* because of the superiority in excess of average fitness associated with the *HBB-β^S^* allele (21).

### Global distribution of the *HBB-*like genes cluster haplotypes

**Figure 1:**
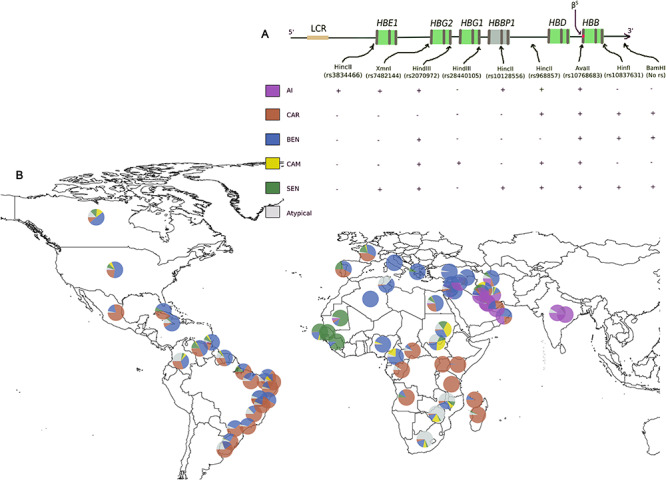
*HBB*-like genes cluster haplotypes: (**A**) Haplotypes defined by presence (+) or absence (−) of specific restriction sites that are associated with the HbS mutation; LCR = locus control region. (**B**) Global distribution of *HBB* haplotypes. CAR = Central African Republic, BEN = Benin, CAM = Cameroon, SEN = Senegal.

The association of combinations of restriction fragment length polymorphic (RFLP) sites, and the *HBB-β^S^* variant results in five ‘classical’ or ‘typical’ *HBB* haplotypes named after the region from where they were first identified (not necessarily where they originated); Senegal (SEN), Benin (BEN), Cameroon (CAM), Central African Republic (CAR) or Bantu and the Arab-India (Fig. 1A). These have served as powerful tools for understanding the evolutionary history of *HBB-β^S^* for over the years. There is considerable genetic diversity within the *HBB-like* gene cluster because of the balancing selection, and this is preserved by a recombination hotspot ~500 bp upstream of *HBB-β^S^* variant. Meanwhile, long-range haplotypes extending across the recombination hotspot in some West African populations (22,23) is consistent with a relatively recent acquisition of the *HBB-β^S^* variant in these populations without enough time for recombination to break them. Generally, the AI, SEN and BEN haplotypes are associated favourable SCD clinical outcomes whereas the CAM and CAR are associated with severe outcomes. However, a recent whole-genome sequencing study that reclassified the classical haplotypes based on linkage disequilibrium, found sub-structuring of the haplotypes that may have confounded previous associations of the haplotypes with SCD clinical severity (1).

Using 60 carefully screened electronic bibliographic records; 28 from recent searches spanning 2015–2020 (Supplementary Information) and 32 from the reference lists of (24) and (25), we made the following observations (Fig. 1B): First, the distribution of the Bantu and BEN haplotypes in the coasts of South America, the Caribbean and North America is consistent with the trans-Atlantic slave trade routes. Second, the prevalence of the Bantu haplotype in Eastern Africa and into Madagascar and the archipelago of Mayotte reflects ancient and recent migrant paths of the Bantu expansion, and more recent migrations. Third, the occurrence of ~7% of the SEN haplotype in the Bengo population of Angola where it was previously absent reflects changing population and disease dynamics that should be important to public health and genetic medicine (26). Fourth, the excess of ‘atypical’ haplotypes (RFPL recombinants that differ from the five major types) in indigenous southern African populations is consistent with low malaria pressure necessary to maintain the conservation of the *HBB* haplotypes in these populations (16). Fifth, Cameroon and Egypt are the only African countries to report all five classical haplotype backgrounds thus far, albeit with low prevalence of the Arab-India haplotype (Supplementary Material, Table S1). Although gene flow may be responsible for this observation, it is likely that *HBB-β^S^* has been in these populations longer, especially in Cameroon (see section The African origin of the *HBB-β^S^* variant).

### The African origin of the *HBB-β^S^* variant

Until recently, there has been no consensus regarding the origin of the *HBB-β^S^* variant. Some studies argued a single origin (unicentric model) (27–29), whereas others posited multiple independent origins via recurrent mutations (multicentric model) coinciding with the adoption of agriculture ~4000–5000 years ago (30,31). It was previously thought that the classical *HBB* haplotypes represented independent origins. However, it is clear that recurrent mutations are less likely to give rise to independent origins of *HBB-β^S^* variant (8,32). Besides, the rate of mutation in haemoglobin genes is too slow to account for multiple independent events of the *HBB-β^S^* variant (33). In addition, gene conversions appear to play no role in the evolution of *HBB-β^S^* (34). Moreover, the absence of the *HBB-β^S^* variant in most indigenous populations outside of Africa means that recurrent mutations are not generating novel *HBB-β^S^* variant. It is now apparent that the different RFLPs used to classify the *HBB-β^S^* haplotypes are not dense or informative enough to capture the extent of the genetic complexity necessary to explain its evolutionary history and clinical significance. Moreover, the specific number and combination of RFLPs used lacks consensus. Genomics techniques have now been used to classify the typical haplotypes based on phased SNP data, meaning that haplotypes can now be predicted in non-homozygous *HBB-β^S^* individuals, thus significantly increasing the power to study the evolutionary history of the *HBB-β^S^* variant (1,35,36).

**Figure 2:**
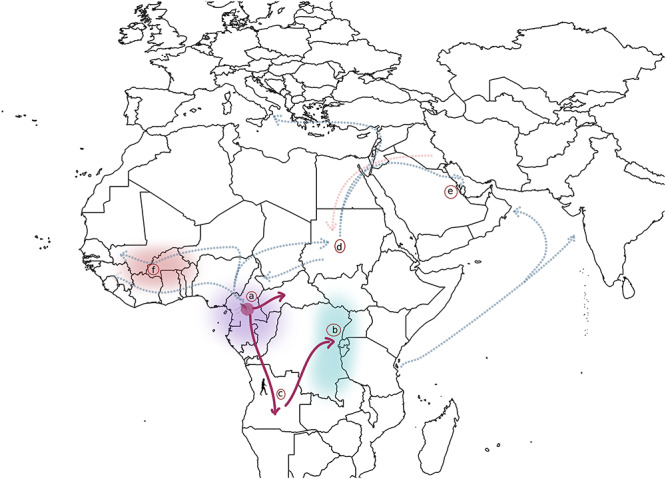
*HBB-β^S^* origin in Africa, population migration dynamics, and evolving research questions. Current data support a single origin of the *HBB-β^S^* variant in central-west Africa in the vicinity of present day Cameroon ~7300 years ago (1), or ~22 000 years ago (14) (**A**); the precise date of occurrence still remains to be determined. This is supported by the following lines of evidence; First, *P. falciparum* diverged from its common ancestor with *P. praefalciparum* 40 000–60 000 years ago in gorillas found around Cameroon (86); the absence of *Plasmodium* infection in eastern gorillas (**B**) further supported this observation. Subterranean malaria pressure probably led to the emergence of *HBB-β^S^* on a CAM *HBB* haplotype background (a) as recently reported (1). It is therefore possible that the occurrence of the *HBB-β^S^ variant* is much older than the current estimates*.* A genetic bottleneck ~5000–6000 years ago and selection of more virulent strains of *P. falciparum,* as a consequence, with a rapid parasite expansion then occurred (38,40), possibly triggering at least one of the waves of the Bantu expansion (**C**). With Bantu expansion, and populations settling in various parts of Africa, it is likely that genomic recombination events within the *HBB-like* genes cluster have generated the other classical *HBB* haplotypes (Fig. 1B), and the regional distributions of these haplotypes have been subsequently shaped by intra African back and forth migratory events, whose sequences and dates are still to be determined (**D**). The distribution of the *HBB-β^S^* variant from Africa into the Mediterranean, the Middle East, and the Indian sub-continent, where the Benin and Indian-Arab haplotype are the most prevalent, could reflect a much recent historical regional migration out of Africa as well as population admixtures, which still need to be properly investigated (**E**). The high prevalence of HbC in West Africa, even though HbS associated genotypes are known to demonstrate excess of average fitness higher than HbC associated genotypes, indicate that *HBB-β^S^* is recent in this West African region (**F**); This needs to be properly investigated from an evolutionary genetic point of view. Lastly, from the time *HBB-β^S^* attained equilibrium in populations [currently estimated at 5000 years ago (1)], it is likely that the pressure because of SCA on the human genome could have contributed to the enrichment of additional adaptive signatures, that still need to be investigated, as suggested by the enrichment of recurrent variants in numerous genes that are relevant to pathophysiology of SCD among patients in Africa (57).

On this basis, one study estimated *HBB-β^S^* to have emerged ~7300 years ago (1), whereas another report estimated *HBB-β^S^* to have originated ~22 000 years ago (14). The mutation was found to have originated in the ancestors of agriculturalists (AGR) from present-day Cameroon (1) (Fig. 2), while only recent (~3000 years ago) acquired by rain forest hunter-gatherers (RHG) following increased gene flow among the two populations in the last ~6000 years (14). There is indeed evidence of increased gene flow among AGR and RHG in the past ~10 000–20 000 years (37). The observation that the CAM haplotype was the closest to the *HBB-β^S^* ancestral haplotype of the five classical haplotypes offers another line of support for a Cameroonian origin (1). Interestingly, data points to the emergence of *P. falciparum* from gorillas some 40 000–60 000 years ago in the western African rainforests around Cameroon (38). More so, the first known close relative of *P. falciparum*—the chimpanzee parasite *P. reichenowi*, which diverged even earlier than the human-gorilla parasites split—was first discovered in Cameroon (39). Thus, there appear to have been subterranean malaria around this region earlier than anywhere else, and predating the adoption of agriculture. More virulent strains that succeeded to establish repeated infections later emerged following a bottleneck in the parasite population ~5000–6000 years ago and rapid population expansions leading to the emergence of the *P. falciparum*-specific erythrocyte invasion protein EBA-175 ~4000 years ago (38). Coinciding with the adoption of agriculture, these events likely ramped up pressure on the human genome, possibly contributing to the Bantu expansion (38,40).

Despite the notable and important recent progresses to estimate the age of *HBB-β^S^*, there have been some shortcomings in the methods used thus far. For instance, Shriner and Rotim (1) assumed a complete recessive lethality of the *HBB-β^S^* (relative fitness = 0) whereas it is known that homozygotes (HbSS) have a relative fitness of 0.2 (recessive lethality = 0.8) (21). Although Laval *et al*. (14) made use of empirical relative fitnesses, they utilized the average *HBB-β^S^* frequency to mean the equilibrium frequency, which is clearly not the case. Given that the estimated *HBB-β^S^* equilibrium frequency (11%) is higher than the average (8.3%) used by Laval *et al*. (14), it implies that the *HBB-β^S^* variant is older than the estimated 22 000 years. Therefore, more research is needed to determine the true age of *HBB-β^S^*.

### SCA and genetic modifiers

Individuals homozygous for *HBB-β^S^* (HbSS) have a relative fitness of 0.2 in malarial environments. Indeed the decreased mortality due to malaria reported in HbSS patients, as compared with homozygous normal individuals (HbAA) (41), is likely to be explained by the by lower parasitaemia in HbSS individuals (42). In addition, a proportion of SCA patients usually live well into their adulthood with few complications and hospitalizations. This long-term survival, without medical interventions, has been associated with the strongest modifiers of SCD, the heredity persistence of ‘non-deletional’ foetal Hb (HbF), a heritable quantitative trait subjected to genetic variations. Three major genetic loci have been associated with higher HbF levels in adults: *Xmn*I-*HBG2* (OMIM: 142250, 11p15.4), *BCL11A* (OMIM: 606557, 2p16.1) and *HBS1L-MYB* (OMIM: 612450, 6q24) (43)*.* The *Xmn*I-*HBG2* (rs7482144) marks the Arab-India and SEN haplotypes and is strongly associated with favourable SCA outcomes (44). *BCL11A,* is an HbF-silencer and whose deactivation—such as with hydroxyurea use—leads to high HbF levels (43,45,46). Targeted variants in *BCL11A* are strongly associated with HbF levels in SCD, including among African patients (47–53). Variants in the intergenic region of *HBSL1* and *MYB* showed even stronger association with HbF levels (47). Other loci recently associated with HbF level include *BCL2L1* (OMIM: 600039, 20q11.21) (54) (which is yet to be replicated in Africa), and *FRMPD4* (OMIM: 300838, Xp22.2) found among patients living with SCD in Tanzania (55). While, it is estimated that the HbF trait is highly heritable (87%) (56), the current known catalogue of variants explain just ~20–50% of the HbF phenotypic variance, indicating that many more variants remain to be uncovered, particularly in African populations, which harbour the highest genetic diversities with usually more extreme phenotypes (57). A particularly interesting new area of research would be to perform genome scans of SCA patients, particularly the long-term survivors, for signatures of adaptation, with specific interest to genomic loci involved in stage-specific expression of globin genes.

A recent whole-exome sequencing study uncovered novel gene modifiers (including *CLCN6* [OMIM: 602726], *OGDHL* [OMIM 617513] and *ATP2B4* [OMIM 108732]) in long-term SCD survivors among SCA patients in Africa who had neither significantly augmented HbF levels, nor alpha-thalassaemia, nor favourable *HBB-β^S^* haplotypes, and yet had significantly fewer complications and hospitalization rates (58). Therefore, although SCD is a monogenic disorder, it is expressed in a polygenic manner such that the tools that are commonly employed for complex disease analysis should be used to yield vital information, to unravel it pathophysiology, and in understanding the contribution of genetic factors to clinical variability in SCD.

## Co-evolution of *HBB-β^S^* and Other Malaria-Resistant Variants: Epistatic and Non-epistatic Interactions

### Co-inheritance of HBB-β^S^ with alpha-thalassaemia

**Figure 3:**
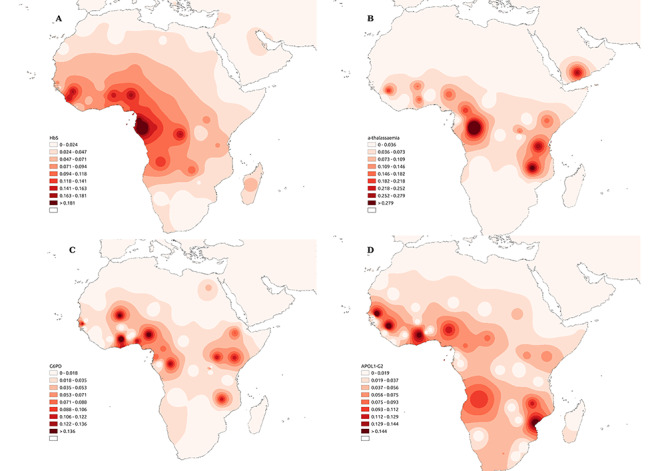
Co-evolution of the *HBB-β^S^* variant and other malaria, and trypanosome associated genes variant in Africa: (**A**) Distribution of the *HBB-β^S^* variant [frequencies adapted from (13)]. (**B**) Distribution of the 3·7 kb alpha-globin gene (*HBA1/HBA2*) deletion [frequencies adapted from (59) and (11)]. (**C**) Distribution of the G6PD deficiency. (**D**) Distribution of the *APOL1* G2 [frequencies adapted from (87)].

The α–thalassaemia trait (α^+^–thalassaemia or −α/−α), is known to protect against severe malaria and like *HBB-β^S^* (Fig. 3A), is thus highly prevalent in malaria endemic areas, particularly the 3·7 kb alpha-globin gene (*HBA1/HBA2*) deletion in Africa (Fig. 3B). In addition, α^+^–thalassaemia is independently associated with favourable SCD clinical outcomes in Africa, whereby an increase in the frequency of the common 3·7 kb alpha-globin gene (*HBA1/HBA2*) deletion leads to low intracellular Hb concentration, reduced haemolytic anaemia, and ultimately delayed onset of clinical manifestations and improved survival (53,59,60). The prevalence of α^+^–thalassaemia in SCA patients shows great age dependence, with increasing prevalence with age, for instance in individuals of African descent in the USA and Cuba (61,62). Thus, there might be adaptive signatures in response to SCA in the *HBA* gene cluster. In Africa, the co-inheritance of α^+^–thalassaemia and SCT, results in reduced severe malaria protection, marking a negative epistatic interaction (63,64). The low prevalence of *HBB-β^S^* in the Mediterranean and its almost completer absence in Oceania (Papua New Guinea) can therefore be explained by very high frequencies of α^+^–thalassaemia (which rises to fixation levels in Papua New Guinea) (65).

### Co-inheritance of HBB-β^S^ with G6PD

Glucose-6-phosphate dehydrogenase (G6PD) deficiency that is heterogeneously associated with malaria protection and thus highly prevalent in malaria-endemic areas (Fig. 3C), appears to be negatively correlated with the SCT in some populations; in India for instance (66). This is expected given that G6PD is required to protect the RBCs from oxidative stress and this anti-oxidant effect protects against haemolytic anaemia in individuals with HbAA affected by malaria, although reports elsewhere are have been conflicting (67,68). However, in some individuals with HbAA, G6PD deficiency is known to increase the risk of severe malaria anaemia (69). Increased risk of severe haemolytic anaemia due to increased oxidative stress therefore exposes SCT carries to harsher clinical outcomes.

### Co-inheritance of HBB-β^S^ with APOE

APOE is encoded be the *APOE* (OMIM: 107741) gene on chromosome 19q13.2 and it is involved in the transfer of plasma cholesterol, an important component of *Plasmodium* parasite metabolism. The ancestral APOE Ɛ4 is associated with elevated plasma cholesterol levels and increased risk of Alzheimer’s disease, and has therefore come under strong negative selection leading to low prevalence in many populations (70). However, in sub-Saharan Africa, where the protein is reported to protect against malaria and hepatitis C, it is observed at high frequencies, particularly in hunter-gatherer populations without any association with Alzheimer’s disease (71). It has been observed from studies involving African children that SCT maintained its protective effect against malaria in children only in the presence of the ancestral APOE Ɛ4 (72). However, the specific interaction between the SCT and APOE has not been extensively explored.

## Co-evolution of *HBB-β^S^* and Other Variants Selected under Specific Non-malaria Environmental Pressures

### Co-inheritance of HBB-β^S^ with variants in APOL1 (trypanosomes pressure)

Association of targeted variants in *APOL1* (G1/G2; rs60910145, rs73885319, rs71785313) with increased risk of nephropathy and end stage renal disease in individuals of African descent has been linked to two coding variants [G1; two nonsynonymous coding variants S342G and I384M, and G2; deletion of two amino acids N388 and Y38] that have been adaptively selected for their protective role against the African trypanosomiasis (73). These *APOL1*gene variants are therefore highly prevalent in sub-Saharan Africa (Fig. 3D) and in African Americans in whom hypertension and kidney disease are highly prevalent. A gene editing study observed a significant exacerbation of nephropathy by the G2 variant under anaemic stress (74). These observations have been largely confirmed by recent on associations of the high-risk variants with exacerbated kidney dysfunction in SCD patients (75,76), and not SCT carriers (77,78).

### Co-inheritance of HBB-β^S^ with variants in OSBPL10 and RXRA (dengue pressure)

A similar increased risk of severe dengue has been associated with SCD (79). Interestingly, the distribution of dengue greatly overlaps that of malaria and SCD, prompting a recent editorial to advocate the implementation of dengue vaccination for children with SCD (80). However, any genetic interaction between variants in *OSBPL10* (OMIM: 606738) and *RXRA* (OMIM: 180245) that are protective against dengue fever (81), and *HBB-β^S^* variant remains to be investigated.

### No HbF protection against malaria

Previous studies had alluded to a possible protective role of HbF against malaria, particularly in neonates (82,83). This spurred interest in a possible epistatic interaction between variants in the HbF-promoting loci and the *HBB-β^S^* variant, among individual living in malaria- endemic regions in Tanzania (48). However, a recent investigation by Archer *et al.*, which cited specific methodological deficiencies in previous studies, found no significant effect of HbF level on *Plasmodium* parasite growth (84). This observation is indeed consistent with clinical trials in Africa, which have consistently shown that hydroxyurea remains relatively safe in malaria-burdened regions (85).

## Conclusion and Perspectives

There is now overwhelming evidence for the single African origin of *HBB-β^S^* variant, likely in the rainforest in the vicinity of the present days Cameroon, at least 7000 years ago. However, the present review has revealed that there is still opportunity to refine the date and, perhaps the location of the emergence of *HBB-β^S^* in Africa. This study emphasized that the classical *HBB-*like genes cluster haplotypes remain a rich source of information in understanding human migration within Africa and out of Africa, by highlighting the distribution of the *HBB* haplotypes and the understanding of the Bantu expansion, as well as the recent migrations, particularly exemplified by the occurrence of ~7% of the Senegal haplotype in Angola that reflects a recent changes in populations migratory dynamics within Africa, and the report of all five classical *HBB* haplotype in Cameroon and Egypt suggests a much longer presence of *HBB-β^S^* in these countries/regions. Thus, it will be interesting to further the studies on the origin of the *HBB-β^S^* variant in these populations.

This review also highlighted the overlapping distribution of *HBB-β^S^* and other malaria resistant variants in *HBB*, *HBA*, *G6PD*, *APOE*, as well as genomic variants selected against trypanosome in *APOL1*, and dengue infections in *OSBPL10* and *RXRA,* for which evolutionary genetic investigations must be urgently performed concurrently, to reveal complex epistasis interactions. Lastly, the present review underlines the need to investigate the enormous pressure that SCA has exerted on the human genome and related adaptively selected genomic loci that are still to be fully uncovered. Therefore, future research should explore the evolutionary history of SCD in relation with other adaptive signatures within the exome, looking at variants of high functional impact, and in the genome, focusing on investigating the missing heritability of the strongest clinical modifiers of SCD i.e. HbF levels. It is also evident that methods used for complex trait and polygenic analyses would yield vital information *HBB-β^S^* genomics, to decipher the panel of genomic information that could enhance genetic medicine practice in Africa, and globally.

## Declaration

A.W. is director of the Sickle in Africa Data Coordinating Centre (SADaCC) based in the University of Cape Town, South Africa. A.W. also directs the Hearing Impairment Genetic Studies in Africa funded by the NIH, USA.

*Conflict of Interest statement*. None declared.

## Supplementary Material

HMGReview_supp_mat_ddab004
