# Supplementary material for: Evolutionary history of sickle-cell mutation: implications for global genetic medicine
Source: Hum Mol Genet. 2021 Jan 18;30(R1):R119–28. doi: 10.1093/hmg/ddab004 (PMC8117455; doi:10.1093/hmg/ddab004)
Supplement: HMGReview_supp_mat_ddab004 [file hmgreview_supp_mat_ddab004.docx]

# Evolutionary history of sickle cell mutation: implications for global genetic medicine

Kevin Esoh^1^, Ambroise Wonkam^1,2*^

^1^Division of Human Genetics, Department of Pathology, University of Cape Town, Cape Town, South Africa.

^2^Department of Medicine, Faculty of Health Sciences, University of Cape Town, Cape Town, South Africa.

***Address correspondence to:**

Ambroise Wonkam, MD, DMedSc, PhD

Division of Human Genetics, Department of Medicine, and

Institute of Infectious Disease and Molecular Medicine

Faculty of Health Sciences, University of Cape Town

Anzio Road, Observatory, 7925, Cape Town, South Africa

Tel: 0027 21 406 63 07

E-mail: [ambroise.wonkam@uct.ac.za](mailto:ambroise.wonkam@uct.ac.za)

# Method

The global georeferenced database of HbS data (1950 – 2015) by Piel et al., (2010 & 2013) was retrieved and complemented by electronic database searches between 2015 – 2020 using the search strategies below and Figure S1.

## **Database**

- **PubMed**: https://pubmed.ncbi.nlm.nih.gov
- **Scopus**: https://www.scopus.com
- **Web of Science**: http://clarivate.libguides.com/webofscienceplatform/alldb

# Search strategy

## ***HbS allele frequency***

(“sickle cell trait” OR "sickle-cell mutation" OR "sickle mutation" OR "sickle cell mutation" OR "Hb S" OR "haemoglobin S" OR "haemoglobin S" OR "haemoglobin mutation" OR "haemoglobin mutation") AND (“sickle cell anaemia”[keyword] OR “haemoglobin S”[keyword] OR “sickle cell”[keyword]) NOT (“case report”[title] OR “with sickle cell disease”[title])

## ***HBB haplotypes***

(“sickle cell trait” OR "sickle-cell mutation" OR "sickle mutation" OR "sickle cell mutation" OR "Hb S" OR "hemoglobin S" OR "haemoglobin S" OR "hemoglobin mutation" OR "haemoglobin mutation" OR "sickle cell") AND haplotype; limited to the last 10 years

**Filters**: Original **journal** **articles** with available **abstract** indexed in **MEDLINE**, **EMBASE**, or present in the **Web of Science core collection** and published in **English** language between **2010** and **2020** were retrieved.

Last search: June 28^th^ 2020, 03:09

Initial screening of the records was based on title and abstract, involving utilizing the following exclusion criteria.

**Exclusion criteria:**

- Case reports and reviews.
- Studies on the prevalence of SCD sub-phenotypes that did not directly measure SCD prevalence
- Studies based on evaluating diagnostic methods without directly measuring the prevalence of HbS genotypes in specific settings.
- Studies with samples unrepresentative of the population e.g. studies on women only, or athletes, or US army soldiers, samples ascertained for hemoglobinopathies etc.
- Studies of HbAS frequency in cases without any matched controls
- Family-based study

The 227 records retained after initial screening were further screened by applying the following inclusion criteria and after manual removal of further duplicates.

## **Inclusion criteria:**

- Studies with direct measure of HbAS

## **Data extraction**

- Demographic: Country, province, age group, sample size
- Genetic: HbAS, HbSS
- Study information: authors and affiliation etc.


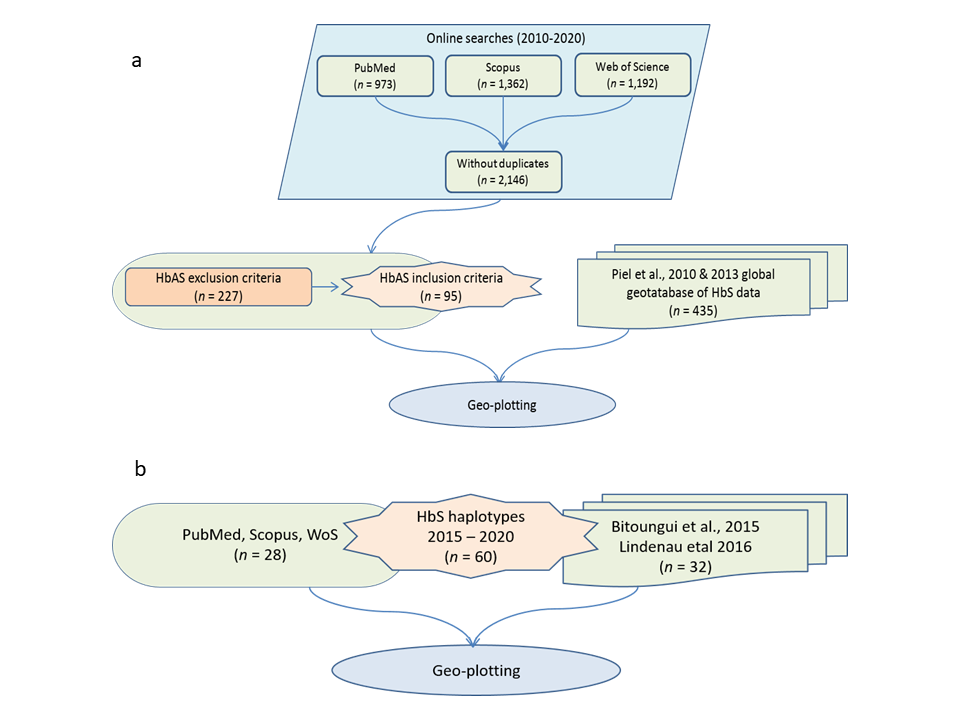


**Figure S1. HbS and HBB haplotypes Search Strategy.** a) Search protocol for HbS global allele frequency in three online electronic bibliographic databases. New searches were integrated with Piel et al. global geodatabase of HbS. b) Search protocol for global HBB distribution. New searches were integrated with Bitoungui et al., 2015 (1) database of global HBB distribution, and Lindenau et al., 2016 (2) database of HBB distribution in Brazil.

# Results

# *HBB* haplotype data were captured in a spreadsheet and organized into Table S1 below.

Table S1. Distribution of βS haplotypes in various World populations

|  | | *Haplotypes (%)* | | | | | |  |
| --- | --- | --- | --- | --- | --- | --- | --- | --- |
| **Continent** | **Country/Region** | **Arab-India** | **CAR/Bantu** | **BEN** | **CAM** | **SEN** | **Atypical** | **Reference** |
| *Africa* | Algeria | 0 | 0 | 100 | 0 | 0 | 0 | (3) |
|  | Angola | 0 | 95.5 | 4.5 | 0 | 0 | 0 | (4) |
|  | Angola, Bengo | 0 | 82.93 | 9.76 | 0 | 7.31 | 0 | (5) |
|  | Cameroon | 0.3 | 0.5 | 73.8 | 19.1 | 0.2 | 6.1 | (1) |
|  | Republic of Congo | 0 | 91 | 0 | 0 | 0 | 9.1 | (6) |
|  | Egypt | 2 | 18.27 | 41.34 | 1 | 5.8 | 5.8 | (7) |
|  | Guinea | 0 | 0 | 11 | 0 | 89 | 0 | (8) |
|  | Kenya | 0 | 98.2 | 1.8 | 0 | 0 | 0 | (9) |
|  | Madagascar | 0 | 91.4 | 0 | 0 | 2.9 | 5.7 | (10) |
|  | Malawi | 0 | 5.7 | 27.1 | 7.1 | 8.7 | 51.4 | (11) |
|  | Mauritania | 5.6 | 4.4 | 8.9 | 0 | 77.8 | 3.3 | (12) |
|  | Nigeria | 0 | 1 | 93.2 | 3.4 | 0 | 2.4 | (13) |
|  | Senegal | 0 | 0 | 0 | 0 | 100 | 0 | (14) |
|  | Senegal, Dakar | 0 | 0.9 | 20.64 | 2.9 | 75.4 | 0.18 | (15) |
|  | South Africa | 0 | 5.1 | 16.6 | 6.4 | 3.9 | 68 | (11) |
|  | Sudan, White Nile | 0 | 2.8 | 29.4 | 35 | 18.2 | 14.6 | (16) |
|  | Sudan, Kordofan | 0 | 2.2 | 21.8 | 30.8 | 12.8 | 32.2 | (17) |
|  | Tanzania | 0 | 100 | 0 | 0 | 0 | 0 | (18) |
|  | Tunisia | 0 | 2.7 | 60.5 | 0 | 0 | 36.7 | (19) |
|  | Uganda | 0 | 99.5 | 0 | 0 | 0.5 | 0 | (20) |
|  | Mayotte Island | 0 | 89.8 | 9.3 | 0.085 | 0 | 0 | (21) |
|  | Zimbabwe | 0 | 3.1 | 12.5 | 15.6 | 3.1 | 65.7 | (11) |
| *Asia* | India | 91.4 | 0 | 0 | 0 | 0 | 8.6 | (22) |
|  | India, Chhattisgarh | 78 | 1 | 2 | 0 | 4 | 15 | (23) |
|  | Bahrain | 89.2 | 5.4 | 2.7 | 0 | 0 | 2.7 | (24) |
|  | Iran, Shiraz | 53.7 | 3.1 | 11.7 | 2.5 | 3.7 | 25.3 | (25) |
|  | Iran, Mazandaran | 23 | 7.7 | 53.9 | 0 | 7.7 | 7.7 | (26) |
|  | Iran, Khuzestan | 38 | 16 | 18 | 16 | 12 | 0 | (27) |
|  | Iraq, Kurdish | 12.5 | 7.8 | 69.5 | 0 | 0 | 10.2 | (28) |
|  | Iraq, Basra | 54.8 | 0 | 19.4 | 0 | 19.4 | 6.5 | (29) |
|  | Jordan | 20 | 0 | 80 | 0 | 0 | 0 | (30) |
|  | Kuwait | 80.8 | 5.6 | 11.2 | 0 | 0 | 2.4 | (31) |
|  | Lebanon | 10 | 15 | 73 | 0 | 0 | 2 | (32) |
|  | Oman | 26.5 | 21.4 | 52.1 | 0 | 0 | 0 | (33) |
|  | West Bank | 0 | 5.1 | 88.1 | 0 | 0 | 6.8 | (34) |
|  | Saudi Arabia, south eastern | 1.6 | 0 | 98.4 | 0 | 0 | 0 | (30) |
|  | Saudi Arabia, eastern province | 82.3 | 0.07 | 0.07 | 1.2 | 4.02 | 4.76 | (35) |
|  | Syria | 33.3 | 0 | 66.7 | 0 | 0 | 0 | (30) |
|  | United Arab Emirates | 52 | 26 | 22 | 0 | 0 | 0 | (36) |
| *America* | Canada | 0 | 11.5 | 49.2 | 13.1 | 13.1 | 13.1 | (18) |
|  | Cuba | 0 | 40.9 | 51 | 0 | 8.1 | 0 | (37) |
|  | Jamaica | 0 | 8.3 | 76 | 0 | 5.2 | 10.5 | (20) |
|  | Mexico | 0 | 78.8 | 18.2 | 0 | 0 | 3 | (38) |
|  | Trinidad | 3.2 | 17.3 | 61.8 | 3.5 | 8.5 | 5.7 | (39) |
|  | USA | 0.8 | 21.6 | 58.9 | 6.1 | 8 | 4.6 | (40) |
|  | Uruguay | 0 | 60 | 20 | 0 | 0 | 20 | (41) |
|  | Brazil, Salvador | 0.4 | 41.6 | 55.2 | 1.2 | 0.4 | 1.2 | (42) |
|  | Brazil, Bahia | 0 | 11.5 | 28.6 | 2.8 | 2.8 | 54.3 | (43) |
|  | Brazil, Rio De Janerio | 0.3 | 73.2 | 19.9 | 0.6 | 0.3 | 5.7 | (44) |
|  | Brazil, Maranhão | 0 | 64.28 | 28.57 | 0 | 0 | 7.15 | (45) |
|  | Brazil, Belém | 0 | 66 | 21.8 | 1.3 | 10.9 | 0 | (46) |
|  | Brazil, Ceará | 0 | 66.2 | 22.1 | 0 | 0 | 11.8 | (47) |
|  | Brazil, São Paulo | 0 | 62.2 | 33.8 | 0 | 0 | 4.1 | (48) |
|  | Brazil, Rio Grande do Sul | 0 | 67.3 | 25 | 0.9 | 0.5 | 6.4 | (2) |
|  | Brazil, Minas Gerais | 0 | 64.8 | 22.1 | 0.8 | 0 | 12.3 | (49) |
|  | Brazil, Bahia | 0 | 48.1 | 45.6 | 0 | 0.63 | 5.63 | (50) |
|  | Brazil, Pernambuco | 0 | 81.1 | 14.2 | 0.8 | 0 | 3.9 | (51) |
|  | Brazil, Rio Grande do Norte | 0 | 75.5 | 11.3 | 6.6 | 0 | 6.6 | (52) |
|  | Brazil, Fortaleza | 0 | 66.2 | 22 | 0 | 0 | 11.8 | (47) |
|  | Brazil, Paraná | 0 | 62 | 32 | 0 | 0 | 6 | (53) |
|  | Colombia | 0.4 | 29.7 | 33.2 | 4.4 | 4.4 | 27.9 | (54) |
|  | Suriname | 0 | 29.9 | 53.2 | 2.6 | 2.6 | 11.7 | (18) |
|  | Venezuela | 0 | 32.2 | 50.8 | 2.3 | 14.1 | 0.6 | (55) |
| *Europe* | Portugal | 0 | 42.4 | 36.4 | 0 | 21.2 | 0 | (4) |
|  | Italy | 0 | 0 | 100 | 0 | 0 | 0 | (56) |
|  | Turkey | 0.5 | 0 | 96.3 | 0 | 0 | 3.3 | (18) |
|  | Greece | 0 | 0 | 92.9 | 0 | 7.1 | 0 | (18) |
|  | France | 0 | 43.2 | 34.6 | 2.7 | 17 | 2.5 | (57) |

# Limitations

Initial screening of articles was based on article title and abstract only. Only a few articles that did not contain HbAS information in title or abstract, which were however sure to contain such information in the main text were retained. Hence some articles with relevant information in the main text were likely missed at this stage.

**References**

1. Bitoungui, V.J.N., Pule, G.D., Hanchard, N., Ngogang, J., Wonkam, A., Ngo Bitoungui, V.J., Pule, G.D., Hanchard, N., Ngogang, J., Wonkam, A. *et al.* (2015) Beta-globin gene haplotypes among cameroonians and review of the global distribution: is there a case for a single sickle mutation origin in Africa? *OMICS*, **19**, 171–9.

2. Lindenau, J.D., Wagner, S.C., de Castro, S.M., Hutz, M.H., Castro, S.M. de, Hutz, M.H. (2016) The effects of old and recent migration waves in the distribution of HBB*S globin gene haplotypes. *Genet Mol Biol*, **39**, 515–23.

3. Pagnier, J., Mears, J.G., Dunda-Belkhodja, O., Schaefer-Rego, K.E., Beldjord, C., Nagel, R.L., Labie, D. (1984) Evidence for the multicentric origin of the sickle cell hemoglobin gene in Africa. *Proc Natl Acad Sci U S A*, **81**, 1771–3.

4. Lavinha, J., Gonçalves, J., Faustino, P., Romão, L., Osório-Almeida, L., Peres, M.J., Picanço, I., Martins, M.C., Ducrocq, R., Labie, D. (1992) Importation route of the sickle cell trait into Portugal: contribution of molecular epidemiology. *Hum Biol*, **64**, 891–901.

5. Borges, E., Tchonhi, C., Couto, C.S.B.B., Gomes, V., Amorim, A., Prata, M.J., Brito, M. (2019) Unusual β-Globin Haplotype Distribution in Newborns from Bengo, Angola. *Hemoglobin*, **43**, 149–54.

6. Mouélé, R., Pambou, O., Feingold, J., Galactéros, F. (2000) alpha-thalassemia in Bantu population from Congo-Brazzaville: its interaction with sickle cell anemia. *Hum Hered*, **50**, 118–25.

7. Abou-Elew, H.H., Youssry, I., Hefny, S., Hashem, R.H., Fouad, N., Zayed, R.A. (2018) β S globin gene haplotype and the stroke risk among Egyptian children with sickle cell disease. *Hematology*, **23**, 362–7.

8. Sow, A., Peterson, E., Josifovska, O., Fabry, M.E., Krishnamoorthy, R., Nagel, R.L. (1995) Linkage-disequilibrium of the senegal haplotype with the beta S gene in the republic of guinea. *Am J Hematol*, **50**, 301–3.

9. Ojwang, P.J., Ogada, T., Beris, P., Hattori, Y., Lanclos, K.D., Kutlar, A., Kutlar, F., Huisman, T.H.J. (1987) Haplotypes and alpha globin gene analyses in sickle cell anaemia patients from Kenya. *Br J Haematol*, **65**, 211–5.

10. Hewitt, R., Krause, A., Goldman, A., Campbell, G., Jenkins, T. (1996) Beta-globin haplotype analysis suggests that a major source of Malagasy ancestry is derived from Bantu-speaking Negroids. *Am J Hum Genet*, **58**, 1303–8.

11. Pule, G.D., Mnica, K., Joubert, M., Mowla, S., Novitsky, N., Wonkam, A. (2017) Burden, genotype and phenotype profiles of adult patients with sickle cell disease in Cape Town, South Africa. *S Afr Med J*, **107**, 149–55.

12. Veten, F.M., Abdelhamid, I.O., Meiloud, G.M., Ghaber, S.M., Salem, M.L., Abbes, S., Houmeida, A.O. (2012) Hb S [β6(A3)Glu→Val, GAG>GTG] and β-globin gene cluster haplotype distribution in Mauritania. *Hemoglobin*, **36**, 311–5.

13. Adekile, A.D., Kitundu, M.N., Gu, L.H., Lanclos, K.D., Adeodu, O.O., Huisman, T.H. (1992) Haplotypes in SS patients from Nigeria; characterization of one atypical beta S haplotype no. 19 (Benin) associated with elevated HB F and high G gamma levels. *Ann Hematol*, **65**, 41–5.

14. Currat, M., Trabuchet, G., Rees, D., Perrin, P., Harding, R.M., Clegg, J.B., Langaney, A., Excoffier, L. (2002) Molecular analysis of the beta-globin gene cluster in the Niokholo Mandenka population reveals a recent origin of the beta(S) Senegal mutation. *Am J Hum Genet*, **70**, 207–23.

15. Tall, F.G., Martin, C., Ndour, E.H.M., Ly, I.D., Renoux, C., Chillotti, L., Veyrenche, N., Connes, P., Gueye, P.M., Diallo, R.N. *et al.* (2017) Genetic Background of the Sickle Cell Disease Pediatric Population of Dakar, Senegal, and Characterization of a Novel Frameshift -Thalassemia Mutation {[}HBB: c.265_266del; p.Leu89Glufs{*}2]. *Hemoglobin*, **41**, 89–95.

16. ELDERDERY, A.Y., MILLS, J., MOHAMED, B.A., Cooper, A.J., MOHAMMED, A.O., ELTIEB, N., Old, J. (2012) Molecular analysis of the β-globin gene cluster haplotypes in a Sudanese population with sickle cell anaemia. *Int J Lab Hematol*, **34**, 262–6.

17. Daak, A.A., Elsamani, E., Ali, E.H., Mohamed, F.A., Abdel-Rahman, M.E., Elderdery, A.Y., Talbot, O., Kraft, P., Ghebremeskel, K., Elbashir, M.I. *et al.* (2016) Sickle cell disease in western Sudan: genetic epidemiology and predictors of knowledge attitude and practices. *Trop Med Int Health*, **21**, 642–53.

18. Oner, C., Dimovski, A.J., Olivieri, N.F., Schiliro, G., Codrington, J.F., Fattoum, S., Adekile, A.D., Oner, R., Yüregir, G.T., Altay, C. (1992) Beta S haplotypes in various world populations. *Hum Genet*, **89**, 99–104.

19. Imen, M., Ikbel, B.M.M., Leila, C., Fethi, M., Amine, Z., Mohamed, B., Salem, A. (2011) Restriction mapping of βS locus among Tunisian sickle-cell patients. *Am J Hum Biol Off J Hum Biol Counc*, **23**, 815–9.

20. Ndugwa, C., Higgs, D., Fisher, C., Hambleton, I., Mason, K., Serjeant, B.E., Serjeant, G.R. (2012) Homozygous sickle cell disease in Uganda and Jamaica a comparison of Bantu and Benin haplotypes. *West Indian Med J*, **61**, 684–91.

21. Muszlak, M., Pissard, S., Badens, C., Chamouine, A., Maillard, O., Thuret, I. (2015) Genetic Modifiers of Sickle Cell Disease: A Genotype-Phenotype Relationship Study in a Cohort of 82 Children on Mayotte Island. *Hemoglobin*, **39**, 156–61.

22. Mukherjee, M.B., Surve, R.R., Gangakhedkar, R.R., Ghosh, K., Colah, R.B., Mohanty, D. (2004) Beta-globin gene cluster haplotypes linked to the betaS gene in western India. *Hemoglobin*, **28**, 157–61.

23. Nongbri, S.R.L., Verma, H.K., Lakkakula, B.V.K.S., Patra, P.K. (2017) Presence of atypical beta globin ( HBB ) gene cluster haplotypes in sickle cell anemia patients of India. *Rev Bras Hematol Hemoter*, **39**, 180–2.

24. Al Arrayed, S., Haites, N. (1995) Features of sickle-cell disease in Bahrain. *East Mediterr Heal J*, **1**.

25. Rahimi, Z., Karimi, M., Haghshenass, M., Merat, A. (2003) Beta-globin gene cluster haplotypes in sickle cell patients from southwest Iran. *Am J Hematol*, **74**, 156–60.

26. Aghajani, F., Mahdavi, M.R.M., Kosaryan, M., Mahdavi, M.R.M., Hamidi, M., Jalali, H. (2016) Identification of β-globin haplotypes linked to sickle hemoglobin (Hb S) alleles in Mazandaran province, Iran. *Genes Genet Syst*, **91**, 311–3.

27. Keikhaei, B., Galehdari, H., Pedram, M., Jaseb, K., Sh, B., Kh, Z., Samadi, B. (2012) Beta-Globin Gene Cluster Haplotypes in Iranian Sickle Cell Patients : Relation to Some Hematologic Parameters. *IJBC*, **4**, 105–10.

28. Al-Allawi, N.A.S., Jalal, S.D., Nerwey, F.F., Al-Sayan, G.O.O., Al-Zebari, S.S.M., Alshingaly, A.A., Markous, R.D., Jubrael, J.M.S., Hamamy, H. (2012) Sickle cell disease in the Kurdish population of northern Iraq. *Hemoglobin*, **36**, 333–42.

29. Yaseen, N., Al-Mamoori, H., Hassan, M. (2020) Sickle ß-globin haplotypes among patients with sickle cell anemia in Basra, Iraq: A cross-sectional study. *Iraqi J Hematol*, **9**, 23.

30. el-Hazmi, M.A., Warsy, A.S., Bashir, N., Beshlawi, A., Hussain, I.R., Temtamy, S., Qubaili, F. (1999) Haplotypes of the beta-globin gene as prognostic factors in sickle-cell disease. *East Mediterr Heal J = La Rev sante la Mediterr Orient = al-Majallah al-sihhiyah li-sharq al-mutawassit*, **5**, 1154–8.

31. Adekile, A.D., Haider, M.Z. (1996) Morbidity, beta S haplotype and alpha-globin gene patterns among sickle cell anemia patients in Kuwait. *Acta Haematol*, **96**, 150–4.

32. Inati, A., Taher, A., Bou Alawi, W., Koussa, S., Kaspar, H., Shbaklo, H., Zalloua, P.A. (2003) Beta-globin gene cluster haplotypes and HbF levels are not the only modulators of sickle cell disease in Lebanon. *Eur J Haematol*, **70**, 79–83.

33. Daar, S., Hussain, H.M., Gravell, D., Nagel, R.L., Krishnamoorthy, R. (2000) Genetic epidemiology of HbS in Oman: multicentric origin for the betaS gene. *Am J Hematol*, **64**, 39–46.

34. Samarah, F., Ayesh, S., Athanasiou, M., Christakis, J., Vavatsi, N. (2009) beta(S)-Globin gene cluster haplotypes in the West Bank of Palestine. *Hemoglobin*, **33**, 143–9.

35. Al-Ali, A.K., Alsulaiman, A., Alzahrani, A.J., Obeid, O.T., Vatte, C.B., Cyrus, C., Alnafie, A.N., Alali, R.A., Alfarhan, M., Mozeleski, B. *et al.* (2020) Prevalence and Diversity of Haplotypes of Sickle Cell Disease in the Eastern Province of Saudi Arabia. *Hemoglobin*, **44**, 78–81.

36. el-Kalla, S., Baysal, E. (1998) Genotype-phenotype correlation of sickle cell disease in the United Arab Emirates. *Pediatr Hematol Oncol*, **15**, 237–42.

37. Muniz, A., Corral, L., Alaez, C., Svarch, E., Espinosa, E., Carbonell, N., di Leo, R., Felicetti, L., Nagel, R.L., Martinez, G. (1995) Sickle cell anemia and beta-gene cluster haplotypes in Cuba. *Am J Hematol*, **49**, 163–4.

38. Magaña, M.T., Ongay, Z., Tagle, J., Bentura, G., Cobián, J.G., Perea, F.J., Casas-Castañeda, M., Sánchez-López, Y.J., Ibarra, B. (2002) Analysis of betaS and betaA genes in a Mexican population with African roots. *Blood Cells Mol Dis*, **28**, 121–6.

39. Jones-Lecointe, A., Smith, E., Romana, M., Gilbert, M.-G., Charles, W.P., Saint-Martin, C., Kéclard, L., Keclard, L., Kéclard, L. (2008) beta-globin gene cluster Haplotypes and alpha-thalassernia in sickle cell disease patients from Trinidad. *Am J Hum Biol*, **20**, 342–4.

40. Crawford, D.C., Caggana, M., Harris, K.B., Lorey, F., Nash, C., Pass, K.A., Tempelis, C., Olney, R.S. (2002) Characterization of beta-globin haplotypes using blood spots from a population-based cohort of newborns with homozygous HbS. *Genet Med*, **4**, 328–35.

41. da Luz, J.A., Sans, M., Kimura, E.M., Albuquerque, D.M., Sonati, M. de F., Costa, F.F. (2006) α-thalassemia, HbS, and β-globin gene cluster haplotypes in two Afro-Uruguayan sub-populations from northern and southern Uruguay. *Genet Mol Biol*, **29**, 595–600.

42. Adorno, E.V., Zanette, Â., Lyra, I., Seixas, M.O., Reis, M.G., Gonçalves, M.S. (2008) Clinical and molecular characteristics of sickle cell anemia in the northeast of Brazil. *Genet Mol Biol*, **31**, 621–5.

43. Nascimento, A.F.F., Oliveira, J.S.S., Silva Junior, J.C.C., Barbosa, A.A.L.A.L., Junior, J.C.S., Barbosa, A.A.L.A.L., Silva Junior, J.C.C., Barbosa, A.A.L.A.L. (2017) Haplotypes and polymorphism in the CCR5 gene in sickle cell disease. *Genet Mol Res*, **16**, DOI: 10.4238/gmr16029675.

44. Okumura, J. V, Silva, D.G.H., Torres, L.S., Belini-Junior, E., Venancio, L.P.R., Carrocini, G.C.S., Nascimento, P.P., Lobo, C.L.C., Bonini-Domingos, C.R. (2019) Atypical β-S haplotypes: classification and genetic modulation in patients with sickle cell anemia. *J Hum Genet*, **64**, 239–48.

45. Alves, A.C., da Silva, V.A.L., Dos Santos, A., Serra, M.B., Marques, F.A., Cruz, S.M.P., Barroso, W.A., de Oliveira, R.A.G. (2020) Sickle cell anemia in the state of Maranhão: a haplotype study. *Ann Hematol*, **99**, 1225–30.

46. Cardoso, G.L., Guerreiro, J.F., Lemos Cardoso, G., Farias Guerreiro, J., Cardoso, G.L., Guerreiro, J.F., Lemos Cardoso, G., Farias Guerreiro, J. (2006) African gene flow to North Brazil as revealed by HBB*S gene haplotype analysis. *Am J Hum Biol*, **18**, 93–8.

47. Da Silva, L.B., Gonçalves, R.P., Rabenhorst, S.H.B. (2009) Analysis of sickle cell anemia haplotypes in Fortaleza reveals the ethnic origins of Ceará state population. *J Bras Patol e Med Lab*, **45**, 115–8.

48. Goncalves, M.S., Nechtman, J.F., Figueiredo, M.S., Kerbauy, J., Arruda, V.R., Sonati, M.F., Saad, S.O., Costa, F.F., Stoming, T.A. (1994) Sickle cell disease in a Brazilian population from Sao Paulo: a study of the beta s haplotypes. *Hum Hered*, **44**, 322–7.

49. Leal, A.S., Martins, P.R.J., Balarin, M.A.S. (2015) Haplotype of the βS-globin cluster in patients with sickle cell anemia at a University Hospital in the Triangulo Mineiro, Minas Gerais. *Rev Bras Hematol Hemoter*, **37**, 140–1.

50. Gonçalves, M.S., Bomfim, G.C., Maciel, E., Cerqueira, I., Lyra, I., Zanette, A., Bomfim, G., Adorno, E. V., Albuquerque, A.L., Pontes, A. *et al.* (2003) βS-Haplotypes in sickle cell anemia patients from Salvador, Bahia, Northeastern Brazil. *Brazilian J Med Biol Res*, **36**, 1283–8.

51. Bezerra, M.A.C., Santos, M.N.N.N., Araújo, A.S., Gomes, Y.M., Abath, F.G.C.C., Bandeira, F.M.G.C.G.C. (2007) Molecular variations linked to the grouping of beta- and alpha-globin genes in neonatal patients with sickle cell disease in the State of Pernambuco, Brazil. *Hemoglobin*, **31**, 83–8.

52. Cabral, C.H.K. (2010) Determinação de haplótipos do gene beta S em pacientes com anemia falciforme. *Rev Bras Hematol Hemoter*, **32**, 491–2.

53. LitsukoTomimatsu Shimauti, E., Humberto Silva, D.G., de Souza, E.M., de Almeida, E.A., Leal, F.P., Bonini-Domingos, C.R. (2015) Prevalence of beta(S)-globin gene haplotypes, alpha-thalassemia (3.7 kb deletion) and redox status in patients with sickle cell anemia in the state of Parana, Brazil. *Genet Mol Biol*, **38**, 316–23.

54. Fong, C., Lizarralde-Iragorri, M.A., Rojas-Gallardo, D., Barreto, G., Alejandra Lizarralde-Iragorri, M., Rojas-Gallardo, D., Barreto, G., Lizarralde-Iragorri, M.A., Rojas-Gallardo, D., Barreto, G. (2013) Frequency and origin of haplotypes associated with the beta-globin gene cluster in individuals with trait and sickle cell anemia in the Atlantic and Pacific coastal regions of Colombia. *Genet Mol Biol*, **36**, 494–7.

55. Arends, A., Alvarez, M., Velázquez, D., Bravo, M., Salazar, R., Guevara, J.M., Castillo, O. (2000) Determination of beta-globin gene cluster haplotypes and prevalence of alpha-thalassemia in sickle cell anemia patients in Venezuela. *Am J Hematol*, **64**, 87–90.

56. Schiliro, G., Samperi, P., Testa, R., Gupta, R.B., Gu, L.H., Huisman, T.H. (1992) Clinical, hematological, and molecular features in Sicilians with Hb S-beta-thalassemia. *Am J Hematol*, **41**, 264–9.

57. Bernaudin, F., Arnaud, C., Kamdem, A., Hau, I., Lelong, F., Epaud, R., Pondarré, C., Pissard, S. (2018) Biological impact of α genes, β haplotypes, and G6PD activity in sickle cell anemia at baseline and with hydroxyurea. *Blood Adv*, **2**, 626–37.
